# Codifying unstructured data: A Natural Language Processing approach to extract rich data from clinical letters

**DOI:** 10.23889/ijpds.v1i1.354

**Published:** 2017-04-19

**Authors:** Arron Lacey, Jane Lyons, Ashley Akbari, Samantha L Turner, Angharad M Walters, Beata Fonferko-Shadrach, Owen Pickrell, Mark I Rees, Ronan A Lyons, David V Ford, Rod M Middleton

**Affiliations:** 1 Swansea University

## Objectives

Electronic healthcare records (EHR) are the main data sources that facilitate epidemiology research. Routinely collected data such as primary and secondary care are now easily linked to produce novel and high impact research. There are, however, rich data locked in the free text of clinical letters that are not otherwise translated into EHRs. It is highly desirable to be able to extract this information to strengthen the body of information in existing EHRs.

The Swansea Collaborative in Analysis of NLP Research (SCANR) group at Swansea University has been established to evaluate the usage of Natural Language Processing platforms for obtaining new clinical data.

To use Clix Enrich to extract SNOMED concepts from a variety of clinical free texts and produce EHRs from the extraction process. 

## Approach

SNOMED concepts contain common items of interest such as diagnosis, medication and symptoms, as well as contextual concepts such as historical reference and negation. Clix Enrich uses the SNOMED dictionary to encode clinical free text (pre-co-ordinated) and find contextually correct SNOMED concepts (post co-ordinated). We used Clix Enrich to extract meaningful clinical terms from MS and Epilepsy consultant letters, as well as presenting complaint fields from a Welsh Emergency Department (ED). 

## Results

We tailored Clix Enrich to extract a wide variety of clinical terms from each source (fourty texts per source) and validated the extraction accuracy with clinical experts in each domain. Clix Enrich was able to accurately extract the correct diagnosis for MS, Epilepsy and ED attendance (100%, 95% and 80%), dosage and frequency of anti-epileptic medication and MS modifying therapy (90%, 100%) and EDDS score (94%). We note a probable source of discrepancy in extraction accuracy between letter sources in the frequency of abbreviated terms, particularly within the presenting complaint field of the ED sample. 

## Conclusion

Clix Enrich can be used to accurately extract SNOMED concepts from clinical letters. The resulting datasets are readily available to link to existing EHRs, and can be linked to EHRs that adopt the SNOMED coding structure, or backward compatible hierarchies. Clix Enrich comes with out-of-the-box extraction methods but the optimum way to extract the correct information would be to build in custom queries, thus requiring clinical expertise to validate extraction.

